# Broken beer bottle as a cause of sigmoid perforation: A summary of causes and predictors in the management of traumatic and non-traumatic colorectal perforation

**DOI:** 10.1016/j.ijscr.2021.106261

**Published:** 2021-08-03

**Authors:** Christian German Ospina-Pérez, Ana Milena Álvarez-Acuña, Lina María López-Álvarez, Rosa María Ospina-Pérez, Ivan David Lozada-Martínez, Sabrina Rahman

**Affiliations:** a Department of Surgery, School of Medicine, University of Cartagena, Cra. 50 #24120, Cartagena, Colombia; b Department of Medicine, Universidad Industrial de Santander, Cl 9 # Cra 27, Bucaramanga, Colombia; c School of Medicine, Fundación Universitaria San Martín, Cl 75 Sur #No 34-50, Sabaneta, Colombia; d Medical and Surgical Research Center, School of Medicine, University of Cartagena, Cra. 50 #24-120, Cartagena, Colombia; e Department of Public Health, Independent University-Bangladesh, Dhaka, Bangladesh

**Keywords:** Foreign bodies, Sigmoid perforation, Colorectal perforation, Colorectal surgery, Case report

## Abstract

**Introduction and importance:**

The presence of foreign bodies at the colorectal level and associated complications is a problem that has become increasingly common in emergency departments. This condition carries high health costs, in addition to having high morbidity and mortality rates, due to the large number of complications such as perforation.

**Case presentation:**

46-year-old male patient, who came to the emergency department of a low-level hospital with a clinical picture of approximately one day of evolution consisting of the violent introduction of a foreign body (bottle) at the anorectal level secondary to aggression in a fight, with subsequent endoluminal rupture.

**Clinical discussion:**

The most recent evidence indicates that the incidence of perforation as a complication of colorectal foreign body introduction is low. However, the presence of profuse bleeding, advanced age, presence of comorbidities and sepsis are predictors of poor prognosis in these cases. In general, perforation secondary to non-traumatic causes is more frequent, being predominantly due to colorectal cancer, ischemia, diverticulitis, inflammatory bowel disease, inadequate use of enema, iatrogenic endoscopy or anorectal manometry or fecal impaction. The presence of unfavorable factors prolongs hospital stay, the risk of reoperation, perianal infection, peritonitis, sepsis and wound infection, generating mortality rates of up to 38%.

**Conclusion:**

Colorectal perforation is more frequent in non-traumatic situations and carries health costs and risk of mortality. Its management depends on hospital aspects, clinical context of the patient and training of health personnel. However, most of the outcomes are favorable.

## Highlights

• The incidence of perforation as a complication of colorectal foreign body introduction is low.
• Colorectal perforation is more frequent in non-traumatic situations.
• The presence of unfavorable factors increases risk of morbidity and mortality.

## 1 Introduction

The presence of foreign bodies at the colorectal level and associated complications is a problem that has become increasingly common in emergency departments [1], [2], [3], [4]. These can be introduced autonomously or by another person, either voluntarily or involuntarily [1], [2], [3], [4]. Those that are introduced autonomously, are related to sexual practices and generally include sex toys such as dildos and regular cylindrical shape; or illegal drugs [1]. Objects introduced by a third party are associated with violent acts such as fights or kidnappings, and are often glass, sticks or bottles [2], [3]. This condition carries high health costs, in addition to having high morbidity and mortality rates, due to the large number of complications that can occur, such as massive bleeding, peritonitis, perforation, obstruction, infection/pelvic sepsis or failure in transanal extraction, requiring recourse to open surgery or laparoscopy, depending on the context clinic and the tools available at the time.

The literature establishes that there is a 28:1 ratio with respect to sex, being more frequent in men than in women, and in young people (20–40 years old) [3]. In low- and middle-income countries where specialized centers or hospitals of high level of complexity are located mainly in large cities [5], its management is complicated due to the fact that most of the traumatic cases of foreign body introduction at the colorectal level occur in rural areas or marginalized areas plagued by violence In low level hospitals, there are no technological tools or trained personnel that can perform transanal extraction or other minimally invasive techniques [5]. Similarly, there are no supplies that can facilitate the management of complications such as perforations, which can quickly deteriorate the patient's clinical condition and lead to death [5].

Much of the literature published so far is limited to sharing the experience and recording the type of object encountered in these cases [2]. Considering the risk of complications and the need to establish early and adequate management, the aim of this manuscript is to report the case of traumatic introduction of a glass bottle at the colorectal level with subsequent sigmoid perforation, and to summarize the causes and prognosis of traumatic and non-traumatic colorectal perforations.

For the summary of causes and predictors in the management of colorectal perforation, a non-systematic search of the literature was performed in the PubMed database over a period of 11 years (2010−2021), with the key terms “Colorectal foreign bodies” and “Colorectal perforation” and synonyms, together with the Boolean operator OR, with the aim of gathering the largest number of related articles; finally obtaining 63 articles. Articles consisting of original studies, case reports, case series and systematic reviews and meta-analyses were included. We excluded those articles that did not present data on causes, predictors and outcomes of colorectal perforation, as well as those that did not have full text available. After the application of these criteria, 12 studies were finally included [6], [7], [8], [9], [10], [11], [12], [13], [14], [15], [16], [17]. This case report followed the SCARE guidelines for its realization [18].

## 2 Presentation of case

46-year-old male patient, who came to the emergency department of a low-level hospital in Colombia, with a clinical picture of approximately one day of evolution consisting of the violent introduction of a foreign body (bottle) at the anorectal level secondary to aggression in a fight, with subsequent endoluminal rupture. The patient reported a sensation of mass, pain and rectorrhagia. As relevant antecedents, he referred diabetes mellitus in treatment with oral antidiabetics and long-standing amputation of a finger on the right hand. The patient had no relevant family history, or any other relevant disorder. On physical examination, the patient was hemodynamically stable, with blood pressure of 128/70 mmHg, heart rate of 86 beats per minute and 15 breaths per minute. In addition, it was detected abdomen with signs of peritoneal irritation and pain in the left iliac fossa.

**Fig. 1:**
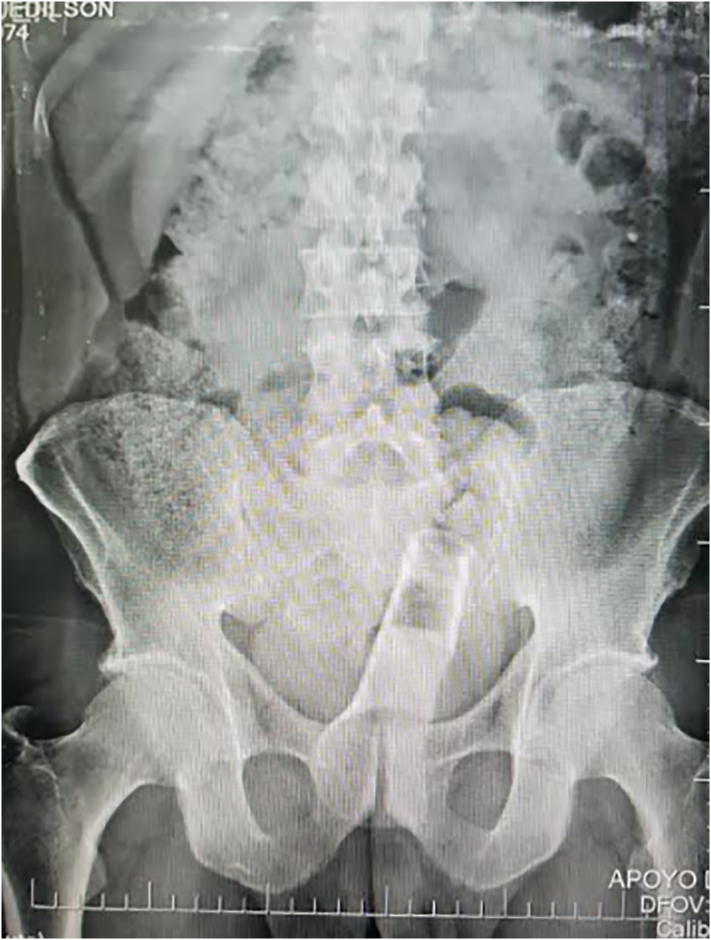
X-ray showing radiopaque image compatible with foreign body in rectum.

**Fig. 2:**
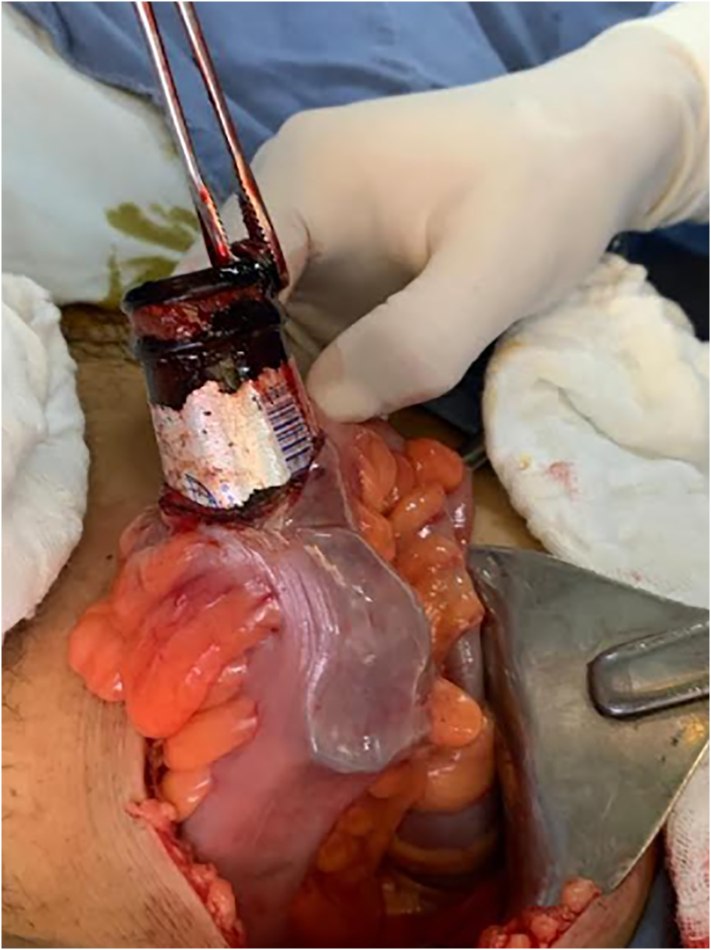
Intraoperative photography. Foreign body at colorectal level.

**Fig. 3:**
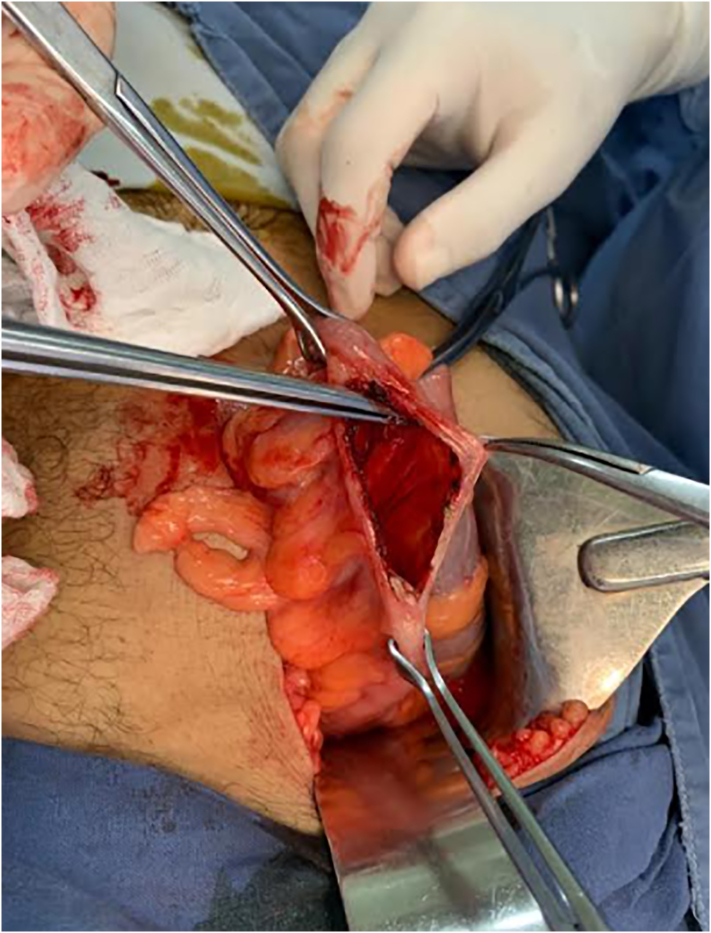
Intraoperative photography. Sigmoid colon enterotomy.

**Fig. 4:**
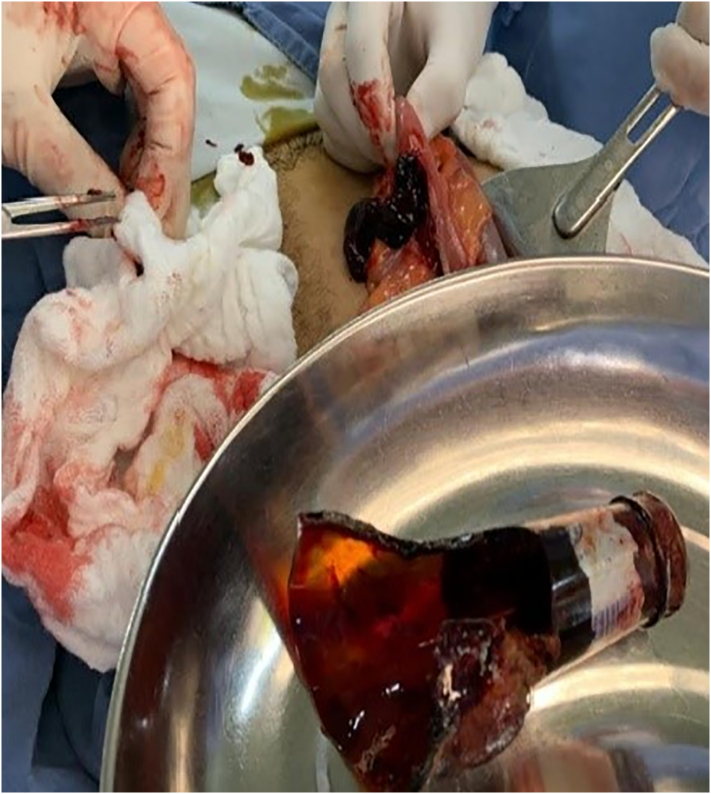
Intraoperative photograph. Extracted foreign body compatible with broken bottle.

Abdominal radiography was performed and a radiopaque image compatible with a foreign body bottle was observed (Fig. 1). Considering the rapid deterioration of the patient, it was decided to perform an exploratory laparotomy with extraction of the foreign body by enterotomy and derivative colostomy by general surgery. A median infraumbilical incision is made where a foreign body is evidenced at the level of the sigmoid colon (Fig. 2), and through enterotomy (Fig. 3) the foreign body is removed in its entirety (Fig. 4). Friable and edematous tissue with perforation was found (Fig. 2), in addition to moderate bleeding, and a loop colostomy was performed. The procedure was completed without complications.

The patient evolved satisfactorily during the postoperative period, without abdominal pain, clean wound, functional colostomy and tolerating the oral route, being discharged after 72 h, with recommendations and alarm signs. The patient came for consultation 7 days later, finding a clean wound with complete resolution of the clinical picture.

## 3 Discussion

The most recent evidence indicates that the incidence of perforation as a complication of colorectal foreign body introduction is low [7], [9], [14], [15], [16]. However, the presence of profuse bleeding, advanced age, presence of comorbidities and sepsis are predictors of poor prognosis in these cases [7], [9], [14], [15], [16]. In general, perforation secondary to non-traumatic causes is more frequent, being predominantly due to colorectal cancer, ischemia, diverticulitis, inflammatory bowel disease, inadequate use of enema, iatrogenic endoscopy or anorectal manometry or fecal impaction [6], [7], [9], [10], [11], [12], [13], [14]. Favorable prognostic factors for a satisfactory outcome in both traumatic and non-traumatic causes are early initiation of surgery and care in a specialized surgical center [6], [12]. The unfavorable prognostic factors are advanced age, requirement of mechanical ventilation, prolonged stay in hospital and intensive care unit, APACHE II (Acute Physiology and Chronic Health Evaluation II) score between 8 and 30, SOFA (Sequential Organ Failure Assessment) score between 0 and 12, DIC (Disseminated Intravascular Coagulation) score between 0 and 8, POSSUM (Physiological and Operative Severity Score for the Enumeration of Mortality and Morbidity) score between 34 and 74, CT (Computerized Tomography) dirty mass volume in cm^3^ (234 ± 211), presence of profuse bleeding, use of conservative treatment, performance of primary anastomosis without a diverting stoma, ASA Grade 3, 4 or 5, chronic steroid use, serum creatinine level > 3.0 mg/dL, disseminated cancer, white blood cell count <3500/mL, low preoperative systolic blood pressure and Hinchey classification IV [6], [7], [8], [9], [10], [11], [12], [13], [14], [15], [16], [17]. Dirty mass is a little-known concept that represents the finding of focal collection of extraluminal fecal matter [19]. It was initially described by Saeki et al. [19], when inquiring about tomographic patterns in colorectal perforation, which had greater sensitivity and specificity than the presence of free air in the peritoneal cavity or gas accumulation in patients with clinical pictures of acute abdomen [19]. Since then, few studies have been devoted to investigate the diagnostic and predictive potential of this radiological sign [20]. However, it has been found to be an unfavorable prognostic factor [7].

The presence of unfavorable factors prolongs hospital stay, the risk of reoperation, perianal infection, peritonitis, sepsis and wound infection, generating mortality rates of up to 38% [6], [7], [8], [9], [10], [11], [12], [13], [14], [15], [16], [17]. Considering the large number of cases and the heterogeneity of outcomes, it is clear that there is still much to be defined in the management of colorectal perforation. Above all, associated with external factors such as sociodemographic condition of the patient and family (which is vital in the follow-up and short-term recovery), hospital infrastructure, time management due to long distances between referral from a hospital of low level of complexity to one of higher level, availability of technological tools and training in the use of minimally invasive techniques (which is vital in the follow-up and recovery in the short term). Therefore, many of the recommendations found in the literature are not extrapolated to surgeons in low- and middle-income countries [5].

However, the initial approach should be the same, using diagnostic imaging to locate and characterize the foreign body (if any, number, size, orientation), or to identify local or regional involvement in case of non-traumatic perforation, to define the patient's clinic status and possible management [1], [3], [4]. For this purpose, abdominal and pelvic radiography, water-soluble enema or tomography can be used [3]. The definitive treatment depends on the cause; in the case of a long and rigid foreign body, transanal extraction can be performed under anesthesia and with appropriate care [3]. However, anxiety and discomfort can generate persistent contraction of the anal sphincter and impede this process. In case of small objects or difficulties in removal, anoscopy or sigmoidoscopy can be performed [1], [3], [4]. It is mandatory to perform proctosigmoidoscopy to check the intestinal integrity and verify that the object has been removed in its entirety. If it is not possible to use any of the previously described tools or they do not allow the removal of the object, it is necessary to proceed with laparoscopic or open surgery, according to the possibilities and the patient's context. In cases of non-traumatic perforation, it is imperative to proceed with surgery to control the risk of peritonitis and sepsis, as well as bleeding [1], [2], [3], [4].

In this context, it is necessary to carry out prospective multicenter studies in all populations and contexts, where the factors that influence the quality and reliability of the outcomes can be evaluated and controlled. Similarly, prognostic factors and outcomes cannot be extrapolated to all situations. Finally, the patient was satisfied with the approach taken and his satisfactory evolution. In contrast to what is currently published in the literature, this manuscript summarizes causes and predictors of the management of traumatic and non-traumatic colorectal perforation, and describes traumatic colorectal perforation secondary to the unintentional introduction of a glass beer bottle by another person in a middle-income country such as Colombia (Table 1).

**Table 1:** Summary of studies reporting causes, predictors and outcomes of traumatic and non-traumatic colorectal perforation.

Authors	Number of patients evaluated	Causes of perforation	Outcome predictors	Outcomes
Matsuoka et al. 2021 [5]	146	Diverticulitis, colorectal cancer, fecal impaction, ischemia	Early onset of surgery (favorable); age, mechanical ventilation, prolonged hospital and ICU stay (unfavorable)	20 deaths/126 survivors
Ishikawa et al. 2021 [6]	58	Diverticulitis, colorectal cancer, ischemia, idiopathic	Mechanical ventilation APACHE II score (8–30), SOFA score (0−12), DIC score (0–8), POSSUM score (34–74), CT dirty mass volume in cm^3^ (234 ± 211) (unfavorable)	13 deaths/45 survivors
Klein et al. 2020 [7]	1	Shattered glass bottle	Profuse bleeding (unfavorable)	Prolonged hospital stay/Alive
Cirocchi et al. 2020 [8]	49	Enema	Conservative treatment (unfavorable)	38% mortality rate
Tsuchiya et al. 2018 [9]	8500	Diverticular disease, colorectal cancer, inflammatory bowel disease, foreign bodies, ischemia, ileus	Performance of primary anastomosis without a diverting Stoma (unfavorable)	Increased number of complications and mortality rate
Lee et al. 2017 [10]	3	Anorectal Manometry	Older age, colorectal cancer (unfavorable)	Alive without complications
Ohki et al. 2017 [11]	10.090	Predominantly colorectal cancer	Specialty hospital care (favorable); ASA Grade 3, 4 or 5, chronic steroid use, serum creatinine level > 3.0 mg/dL, disseminated cancer, white blood cell count <3500/mL (unfavorable)	Mortality of 11.36%.
Hsu et al. 2017 [12]	423	Diverticulitis, ischemic proctocolitis, colorectal cancer, iatrogenic injury, deep ulceration, trauma	Age older than 70 years, presence of more than 3 comorbidities, preoperative sepsis or septic shock, reoperation for uncontrolled peritonitis, cancer or ischemic proctocolitis as etiology, Hinchey classification IV (unfavorable)	Very high mortality
Yamamoto et al. 2015 [13]	108	Diverticulosis, colorectal cancer, fecal impaction, inflammatory bowel disease, trauma	Older age, low preoperative systolic blood pressure (unfavorable)	12% mortality rate
Yildiz et al. 2013 [14]	30	Foreign body	–	Wound infection and perianal infection
Boettcher et al. [15]	1	Broomstick	–	Patient alive without complications
Kurer et al. [16]	13	Foreign body	–	Sepsis/peritonitis - only one patient died

APACHE II: acute physiology and chronic health evaluation; CT: Computerized tomography; DIC: disseminated intravascular coagulation; POSSUM: physiological and operative severity score for the enumeration of mortality and morbidity; SOFA: sequential organ failure assessment; WBC: White blood cells.

## 4 Conclusion

The introduction of foreign bodies at the colorectal level is a condition that has become frequent, mainly due to the voluntary use of sex toys, predominantly in young men. Colorectal perforation is more frequent in non-traumatic situations and carries health costs and risk of mortality. Its management depends on hospital aspects, clinical context of the patient and training of health personnel. However, most of the outcomes are favorable.

## Abbreviations

APACHE II: Acute Physiology and Chronic Health Evaluation II
CT: Computerized Tomography
DIC: Disseminated Intravascular Coagulation
POSSUM: Physiological and Operative Severity Score for the Enumeration of Mortality and Morbidity
SOFA: Sequential Organ Failure Assessment
WBC: White Blood Cells

## Sources of funding

None declared.

## Ethical approval

Hospital exempts ethics approval for reported cases.

## Consent written

Written informed consent was obtained from the patient for publication of this case report and accompanying images. A copy of the written consent is available for review by the Editor-in-Chief of this journal on request.

## CRediT authorship contribution statement

All authors equally contributed to the analysis and writing of the manuscript.

## Research registration

Not applicable.

## Guarantor

Sabrina Rahman. Department of Public Health, Independent University-Bangladesh, Dhaka, Bangladesh. sabrinaemz25@gmail.com.

## Provenance and peer review

Not commissioned, externally peer-reviewed.

## Declaration of competing interest

The authors declare that they have no known competing financial interests or personal relationships that could have appeared to influence the work reported in this paper.
